# (2,2′-Bipyridine-κ^2^ *N*,*N*′)diiodido­palladium(II)

**DOI:** 10.1107/S1600536809047771

**Published:** 2009-11-14

**Authors:** Kwang Ha

**Affiliations:** a School of Applied Chemical Engineering, The Research Institute of Catalysis, Chonnam National University, Gwangju 500-757, Republic of Korea

## Abstract

The asymmetric unit of the title complex, [PdI_2_(C_10_H_8_N_2_)], contains one half of the formula unit. The Pd^2+^ ion is located on a twofold rotation axis and is four-coordinated in a slightly distorted square-planar environment by two N atoms of the chelating 2,2′-bipyridine ligand and two iodide ions. The compound displays inter­molecular π–π inter­actions between the pyridine rings of the ligand, the shortest centroid–centroid distance being 4.220 (4) Å.

## Related literature

For the crystal structures of [Pd*X*_2_(bipy)] (bipy = 2,2′-bipyridine; *X* = Cl or Br), see: Maekawa *et al.* (1991 ▶); Smeets *et al.* (1997 ▶). For the crystal structures of [Pd*X*_2_(bipy)]·CH_2_Cl_2_ (*X* = Cl or Br), see: Vicente *et al.* (1997 ▶); Kim *et al.* (2009 ▶); Kim & Ha (2009 ▶).
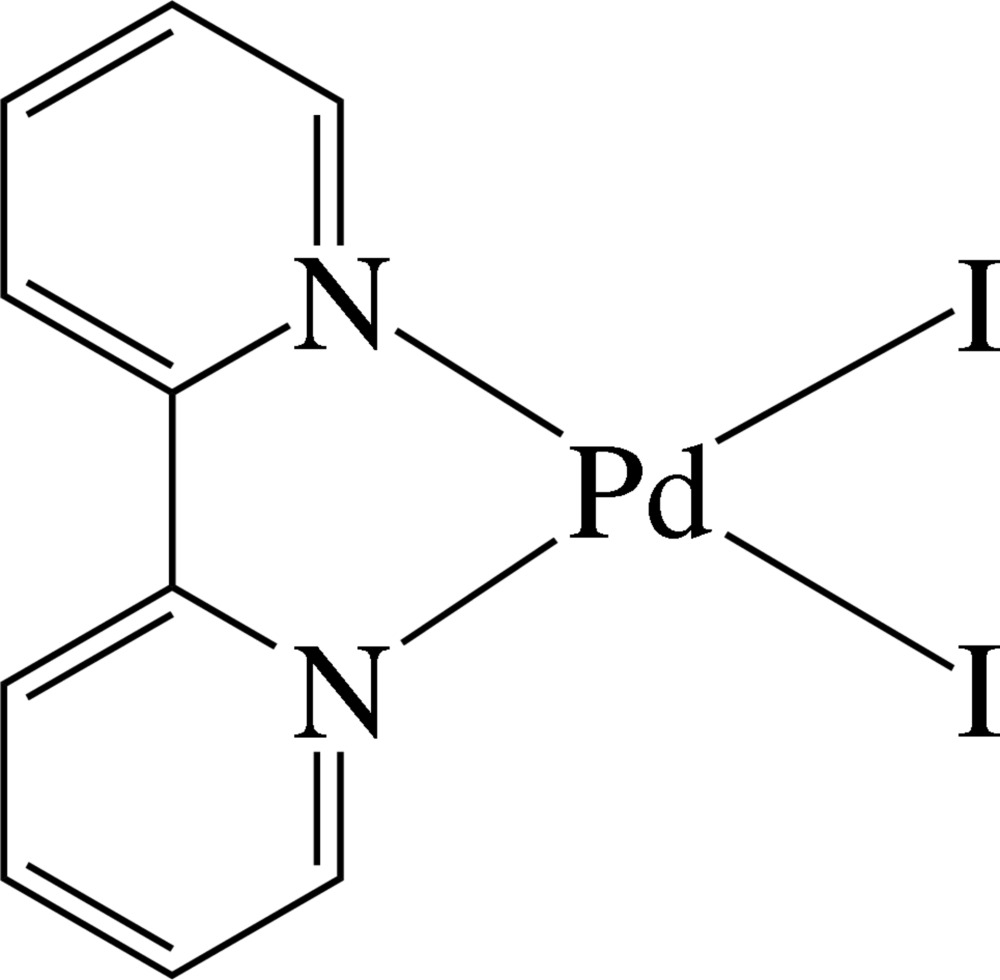


## Experimental

### 

#### Crystal data

[PdI_2_(C_10_H_8_N_2_)]
*M* *_r_* = 516.38
Monoclinic, 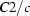

*a* = 17.232 (4) Å
*b* = 9.8273 (19) Å
*c* = 7.6868 (15) Å
β = 111.438 (3)°
*V* = 1211.6 (4) Å^3^
*Z* = 4
Mo *K*α radiation
μ = 6.60 mm^−1^
*T* = 293 K
0.25 × 0.05 × 0.05 mm

#### Data collection

Bruker SMART 1000 CCD diffractometer
Absorption correction: multi-scan (**SADABS**; Bruker, 2000 ▶) *T*_min_ = 0.139, *T*_max_ = 0.719
3458 measured reflections
1240 independent reflections
1049 reflections with *I* > 2σ(*I*)
*R*_int_ = 0.025

#### Refinement

*R*[*F* ^2^ > 2σ(*F* ^2^)] = 0.032
*wR*(*F* ^2^) = 0.071
*S* = 1.06
1240 reflections
69 parameters
H-atom parameters constrained
Δρ_max_ = 0.60 e Å^−3^
Δρ_min_ = −0.65 e Å^−3^

### 

Data collection: *SMART* (Bruker, 2000 ▶); cell refinement: *SAINT* (Bruker, 2000 ▶); data reduction: *SAINT*; program(s) used to solve structure: *SHELXS97* (Sheldrick, 2008 ▶); program(s) used to refine structure: *SHELXL97* (Sheldrick, 2008 ▶); molecular graphics: *ORTEP-3* (Farrugia, 1997 ▶) and *PLATON* (Spek, 2009 ▶); software used to prepare material for publication: *SHELXL97*.

## Supplementary Material

Crystal structure: contains datablocks global, I. DOI: 10.1107/S1600536809047771/is2486sup1.cif

Structure factors: contains datablocks I. DOI: 10.1107/S1600536809047771/is2486Isup2.hkl

Additional supplementary materials:  crystallographic information; 3D view; checkCIF report

## Figures and Tables

**Table:** 

Pd1—N1	2.076 (4)
Pd1—I1	2.5704 (6)

**Table:** 

N1—Pd1—N1^i^	79.4 (2)

Symmetry code: (i) 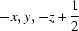.

**Fig. 1.**
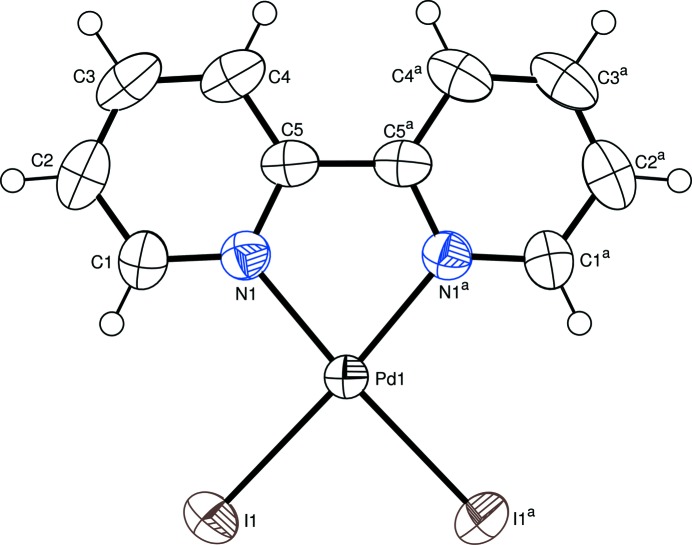
The structure of the title complex, with displacement ellipsoids drawn at the 50% probability level for non-H atoms [Symmetry code: (a) -x, y, 1/2 - z].

**Fig. 2.**
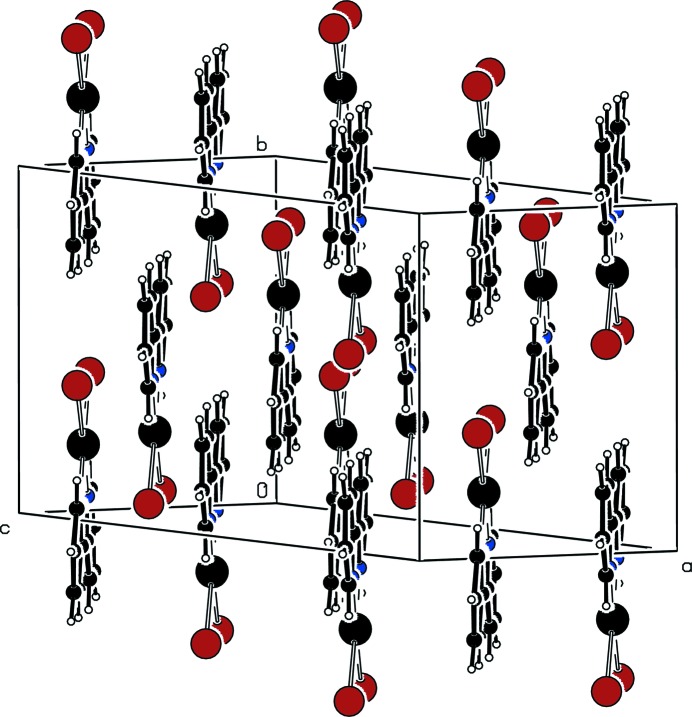
Crystal packing of the title complex.

## supplementary crystallographic information

### Comment

The title complex, [PdI_2_(bipy)] (where bipy is 2,2'-bipyridine, C_10_H_8_N_2_), is isomorphous with [PdBr_2_(bipy)] (Smeets *et al.*, 1997), whereas [PdCl_2_(bipy)] crystallized in the orthorhombic space group *C*222_1_ (Maekawa *et al.*, 1991).

The asymmetric unit of the title complex contains one half of the formula unit. The complex is disposed about a twofold rotation axis through Pd atom with the special position at (0, *y*, 1/4) (Wyckoff letter *e*). The Pd^2+^ ion is four-coordinated in a slightly distorted square-planar environment by two N atoms of the chelating 2,2'-bipyridine ligand and two iodide ions (Fig. 1). The main contribution to the distortion is the tight N1—Pd1—N1*^a^* [symmetry code: (*a*) -*x*, *y*, 1/2 - *z*] chelate angle [79.4 (2)°], which results in non-linear *trans* arrangement [<N1—Pd1—I1*^a^* = 175.85 (12)°]. The complex displays intermolecular π-π interactions between adjacent pyridine rings of the lignad (the symmetry operation for second plane *x*, -*y*, -1/2 + *z*), with a shortest centroid-centroid distance of 4.220 (4) Å (Fig. 2).

### Experimental

To a solution of Na_2_PdCl_4_ (0.1991 g, 0.677 mmol) in H_2_O (20 ml) were added KI (1.1230 g, 6.765 mmol) and 2,2'-bipyridine (0.1057 g, 0.677 mmol), and refluxed for 3 h. The precipitate obtained was separated by filtration, and washed with water and acetone, and dried at 70 °C, to give a red-brown powder (0.2999 g). Crystals suitable for X-ray analysis were obtained by slow evaporation from a CH_3_CN solution.

### Refinement

H atoms were positioned geometrically and allowed to ride on their respective parent atoms [C—H = 0.93 Å and *U*_iso_(H) = 1.2*U*_eq_(C)].

### Crystal data

**Table:**

[PdI_2_(C_10_H_8_N_2_)]	*F*(000) = 936
*M**_r_* = 516.38	*D*_x_ = 2.831 Mg m^−^^3^
Monoclinic, *C*2/*c*	Mo *K*α radiation, λ = 0.71073 Å
Hall symbol: -C 2yc	Cell parameters from 550 reflections
*a* = 17.232 (4) Å	θ = 2.4–24.4°
*b* = 9.8273 (19) Å	µ = 6.60 mm^−^^1^
*c* = 7.6868 (15) Å	*T* = 293 K
β = 111.438 (3)°	Needle, brown
*V* = 1211.6 (4) Å^3^	0.25 × 0.05 × 0.05 mm
*Z* = 4	

### Data collection

**Table:**

Bruker SMART 1000 CCD diffractometer	1240 independent reflections
Radiation source: fine-focus sealed tube	1049 reflections with *I* > 2σ(*I*)
graphite	*R*_int_ = 0.025
φ and ω scans	θ_max_ = 26.4°, θ_min_ = 2.4°
Absorption correction: multi-scan (*SADABS*; Bruker, 2000)	*h* = −21→13
*T*_min_ = 0.139, *T*_max_ = 0.719	*k* = −11→12
3458 measured reflections	*l* = −9→9

### Refinement

**Table:**

Refinement on *F*^2^	Primary atom site location: structure-invariant direct methods
Least-squares matrix: full	Secondary atom site location: difference Fourier map
*R*[*F*^2^ > 2σ(*F*^2^)] = 0.032	Hydrogen site location: inferred from neighbouring sites
*wR*(*F*^2^) = 0.071	H-atom parameters constrained
*S* = 1.06	*w* = 1/[σ^2^(*F*_o_^2^) + (0.0328*P*)^2^] where *P* = (*F*_o_^2^ + 2*F*_c_^2^)/3
1240 reflections	(Δ/σ)_max_ < 0.001
69 parameters	Δρ_max_ = 0.60 e Å^−^^3^
0 restraints	Δρ_min_ = −0.65 e Å^−^^3^

### Special details

**Table:**

Geometry. All e.s.d.'s (except the e.s.d. in the dihedral angle between two l.s. planes) are estimated using the full covariance matrix. The cell e.s.d.'s are taken into account individually in the estimation of e.s.d.'s in distances, angles and torsion angles; correlations between e.s.d.'s in cell parameters are only used when they are defined by crystal symmetry. An approximate (isotropic) treatment of cell e.s.d.'s is used for estimating e.s.d.'s involving l.s. planes.
Refinement. Refinement of *F*^2^ against ALL reflections. The weighted *R*-factor *wR* and goodness of fit *S* are based on *F*^2^, conventional *R*-factors *R* are based on *F*, with *F* set to zero for negative *F*^2^. The threshold expression of *F*^2^ > σ(*F*^2^) is used only for calculating *R*-factors(gt) *etc*. and is not relevant to the choice of reflections for refinement. *R*-factors based on *F*^2^ are statistically about twice as large as those based on *F*, and *R*- factors based on ALL data will be even larger.

### Fractional atomic coordinates and isotropic or equivalent isotropic displacement parameters (Å^2^)

**Table:**

	*x*	*y*	*z*	*U*_iso_*/*U*_eq_	
Pd1	0.0000	−0.19021 (5)	0.2500	0.03813 (17)	
I1	0.10045 (3)	−0.37899 (4)	0.43052 (5)	0.06273 (19)	
N1	0.0755 (3)	−0.0277 (4)	0.3822 (6)	0.0431 (10)	
C1	0.1524 (4)	−0.0339 (6)	0.5132 (7)	0.0546 (14)	
H1	0.1767	−0.1188	0.5499	0.066*	
C2	0.1966 (4)	0.0802 (7)	0.5952 (9)	0.0655 (17)	
H2	0.2495	0.0725	0.6871	0.079*	
C3	0.1617 (4)	0.2050 (6)	0.5399 (9)	0.0656 (18)	
H3	0.1902	0.2837	0.5944	0.079*	
C4	0.0840 (4)	0.2128 (6)	0.4029 (9)	0.0611 (17)	
H4	0.0597	0.2972	0.3627	0.073*	
C5	0.0418 (4)	0.0954 (5)	0.3248 (8)	0.0446 (12)	

### Atomic displacement parameters (Å^2^)

**Table:**

	*U*^11^	*U*^22^	*U*^33^	*U*^12^	*U*^13^	*U*^23^
Pd1	0.0378 (3)	0.0339 (3)	0.0385 (3)	0.000	0.0091 (2)	0.000
I1	0.0636 (3)	0.0471 (3)	0.0633 (3)	0.01213 (18)	0.0064 (2)	0.00869 (16)
N1	0.043 (2)	0.043 (2)	0.045 (2)	−0.002 (2)	0.018 (2)	−0.0005 (19)
C1	0.046 (3)	0.062 (4)	0.050 (3)	−0.003 (3)	0.012 (3)	−0.004 (3)
C2	0.050 (4)	0.087 (5)	0.056 (4)	−0.019 (4)	0.016 (3)	−0.015 (3)
C3	0.065 (4)	0.060 (4)	0.082 (4)	−0.029 (4)	0.039 (4)	−0.028 (3)
C4	0.067 (4)	0.050 (3)	0.074 (4)	−0.018 (3)	0.036 (4)	−0.015 (3)
C5	0.050 (3)	0.039 (3)	0.056 (3)	−0.001 (2)	0.033 (3)	−0.002 (2)

### Geometric parameters (Å, °)

**Table:**

Pd1—N1	2.076 (4)	C2—C3	1.364 (9)
Pd1—N1^i^	2.076 (4)	C2—H2	0.9300
Pd1—I1^i^	2.5704 (6)	C3—C4	1.370 (9)
Pd1—I1	2.5704 (6)	C3—H3	0.9300
N1—C1	1.341 (7)	C4—C5	1.378 (7)
N1—C5	1.345 (6)	C4—H4	0.9300
C1—C2	1.372 (8)	C5—C5^i^	1.480 (12)
C1—H1	0.9300		
			
N1—Pd1—N1^i^	79.4 (2)	C3—C2—C1	119.0 (6)
N1—Pd1—I1^i^	175.85 (12)	C3—C2—H2	120.5
N1^i^—Pd1—I1^i^	96.48 (12)	C1—C2—H2	120.5
N1—Pd1—I1	96.48 (12)	C2—C3—C4	119.0 (6)
N1^i^—Pd1—I1	175.85 (12)	C2—C3—H3	120.5
I1^i^—Pd1—I1	87.61 (3)	C4—C3—H3	120.5
C1—N1—C5	118.5 (5)	C3—C4—C5	120.0 (6)
C1—N1—Pd1	127.1 (4)	C3—C4—H4	120.0
C5—N1—Pd1	114.4 (4)	C5—C4—H4	120.0
N1—C1—C2	122.6 (6)	N1—C5—C4	120.9 (6)
N1—C1—H1	118.7	N1—C5—C5^i^	115.9 (3)
C2—C1—H1	118.7	C4—C5—C5^i^	123.2 (4)
			
N1^i^—Pd1—N1—C1	−178.7 (6)	C2—C3—C4—C5	0.8 (9)
I1—Pd1—N1—C1	2.1 (5)	C1—N1—C5—C4	−2.1 (8)
N1^i^—Pd1—N1—C5	0.5 (3)	Pd1—N1—C5—C4	178.6 (4)
I1—Pd1—N1—C5	−178.7 (3)	C1—N1—C5—C5^i^	177.9 (6)
C5—N1—C1—C2	2.3 (9)	Pd1—N1—C5—C5^i^	−1.4 (7)
Pd1—N1—C1—C2	−178.5 (4)	C3—C4—C5—N1	0.6 (9)
N1—C1—C2—C3	−0.9 (10)	C3—C4—C5—C5^i^	−179.4 (6)
C1—C2—C3—C4	−0.6 (10)		

Symmetry codes: (i) −*x*, *y*, −*z*+1/2.
